# A Mechanistic Model of Intermittent Gastric Emptying and Glucose-Insulin Dynamics following a Meal Containing Milk Components

**DOI:** 10.1371/journal.pone.0156443

**Published:** 2016-06-02

**Authors:** Priska Stahel, John P. Cant, Jayden A. R. MacPherson, Harma Berends, Michael A. Steele

**Affiliations:** 1 Department of Animal Biosciences, University of Guelph, Guelph, Ontario, Canada; 2 Department of Agriculture, Food and Nutritional Services, University of Alberta, Edmonton, Alberta, Canada; 3 Trouw Nutrition R&D, Boxmeer, North Brabant, the Netherlands; Sonoma State University, UNITED STATES

## Abstract

To support decision-making around diet selection choices to manage glycemia following a meal, a novel mechanistic model of intermittent gastric emptying and plasma glucose-insulin dynamics was developed. Model development was guided by postprandial timecourses of plasma glucose, insulin and the gastric emptying marker acetaminophen in infant calves fed meals of 2 or 4 L milk replacer. Assigning a fast, slow or zero first-order gastric emptying rate to each interval between plasma samples fit acetaminophen curves with prediction errors equal to 9% of the mean observed acetaminophen concentration. Those gastric emptying parameters were applied to glucose appearance in conjunction with minimal models of glucose disposal and insulin dynamics to describe postprandial glycemia and insulinemia. The final model contains 20 parameters, 8 of which can be obtained by direct measurement and 12 by fitting to observations. The minimal model of intestinal glucose delivery contains 2 gastric emptying parameters and a third parameter describing the time lag between emptying and appearance of glucose in plasma. Sensitivity analysis of the aggregate model revealed that gastric emptying rate influences area under the plasma insulin curve but has little effect on area under the plasma glucose curve. This result indicates that pancreatic responsiveness is influenced by gastric emptying rate as a consequence of the quasi-exponential relationship between plasma glucose concentration and pancreatic insulin release. The fitted aggregate model was able to reproduce the multiple postprandial rises and falls in plasma glucose concentration observed in calves consuming a normal-sized meal containing milk components.

## Introduction

The mathematical simulation of glucose-insulin dynamics in response to a meal is of great value for decision support related to management of plasma glucose in animals under our care, including domestic species and human patients. The classification of foods according to their effect on the incremental area under the curve of plasma glucose concentrations after ingestion, the so-called glycemic index, was developed to assist in glycemia management [1]. The glycemic response is not just a characteristic of foods, however. Upon consumption of a meal, the ability to dispose of the absorbed glucose, and thus minimize postprandial hyperglycemia and its potentially negative consequences, is dependent on the subject’s pancreatic responsiveness, insulin sensitivity and glucose effectiveness. Absorbed glucose has a varying ability to stimulate pancreatic insulin secretion across individuals, with type 1 diabetes being an extreme case in which insulin is only minimally secreted or not at all. The circulating insulin acts on the liver, muscle and adipose tissues to decrease hepatic glucose production and increase peripheral glucose disposal, respectively. In addition, glucose can stimulate its own disposal via a mass-action effect on influx into various tissues. Impairments in any of these three factors of pancreatic responsiveness, insulin sensitivity or glucose effectiveness can exacerbate postprandial hyperglycemia, a hallmark of metabolic syndrome and type 2 diabetes. The minimal glucose and insulin models of Bergman *et al*. [2] and Toffolo *et al*. [3] describe all three of these contributions to glucose disposal following an intravenous glucose dose.

Aside from the prominent role of insulin, appearance of glucose in plasma following ingestion of a carbohydrate laden meal is dependent on the rate of gastric emptying. In the early phase of meal consumption, the proximal gastric wall distends and relaxes to accommodate the increased volume. This is quickly followed by tonic contractions of the proximal stomach to push contents towards the distal end where large particles are degraded, aided by the action of peristaltic contractions, and from which small particles and liquids can be pushed into the duodenum through the pyloric sphincter [4]. Liquids display exponential gastric emptying proportional to the gastric volume and without an initial lag, while solids show biphasic gastric emptying with a lag [4]. The intestine responds to early sampling of stomach contents following emptying by altering gut peptide secretion, dependent on the characteristics of the meal. This altered gut peptide profile influences neuro-hormonal control of gastrointestinal function and gastric emptying. Overall, postprandial gastric emptying is a function of meal composition, volume, osmolality and caloric load [4,5].

Equations to simulate gastric emptying have been incorporated into models to interpret plasma and glucose curves following an oral glucose tolerance test [6,7] but we are aware of only one effort to include gastric emptying equations in the simulation of glycemic responses to a normal-sized meal [8,9]. To simulate the pattern of plasma glucose concentrations following a meal, Dalla Man *et al*. [9] combined a gastric emptying/absorption model of 9 parameters with a glucose-insulin model of 26 parameters, where the 4 components of gastric emptying/absorption, glucose utilization, endogenous glucose production, and pancreatic insulin secretion were each fitted separately from tracer flux and plasma concentration data. Part of the difficulty with modelling gastric emptying is that outflow from the stomach is intermittent, which produces an erratic timecourse. We have previously used models of 2 to 4 parameters to describe the intermittent appearance of acetaminophen (Ac) in plasma following an oral dose [10]. Acetaminophen is very slowly absorbed from the stomach but rapidly absorbed in the proximal small intestine [11] so its appearance in plasma can be used to estimate gastric emptying rate [10].

In this paper, we present the development and evaluation of a novel mechanistic model that incorporates glucose-insulin dynamics of MINMOD [12] with an intermittent gastric emptying model [10] to predict glycemic responses to a milk-based meal in infant cattle. Prior to development of the ruminant habit, the abomasum of pre-weaned calves functions like the glandular stomach of monogastric animals [13] The carbohydrate in milk is the liquid-associated disaccharide lactose, made up of galactose and glucose moieties. It is hydrolyzed by intestinal lactase (EC 3.2.1.108) and approximately 90% of the absorbed galactose is converted to glycogen in the liver [14,15] while approximately 90% of the absorbed glucose enters the peripheral circulation as free glucose [7,9]. We used the aggregate model to evaluate the importance of gastric emptying to postprandial glycemia, relative to the parameters of insulin release and glucose disposal.

## Model Structure

### Database

Four Holstein-Friesian female calves, housed individually in wheat-straw bedded hutches, at the Trouw Nutrition Ruminant Research facility (Boxmeer, The Netherlands) were maintained on either 4 or 8L of milk replacer (150 g/L dry matter; 24% crude protein, 18% crude fat, and 45.2% lactose on a dry basis; Trouw Nutrition, Deventer, The Netherlands) given twice daily from day 8 to 7 weeks of age. At 4 and 7 weeks of age, calves were provided their morning meal of milk replacer containing 150 mg/kg BW^0.75^ Ac as a gastric emptying marker. Blood samples were taken from a jugular vein catheter at -30, 30, 60, 90, 120, 150, 180, 210, 240, 300, 360 and 420 minutes relative to the meal and plasma was analyzed for cAc_P_, cGl_P_ and cIn_P_. The -30-min time-point was used as the 0-min time-point in the model (S1 file). The meals of 2 and 4 L contained 136 and 271 g glucose, respectively, producing a range of 1.8 to 5.1 g/kg BW. Postprandial timecourses of plasma concentrations of acetaminophen (cAc_P_), glucose (cGl_P_) and insulin (cIn_P_) in calves at 4 and 7 weeks of age from MacPherson *et al*. [16] were used to guide model development. Procedures complied with the Dutch Law on Experimental Animals, and the ETS123 (Council of Europe 1985 and the 86/609/EEC Directive) and were approved by the Animal Care and Use Committee from Utrecht University.

**Fig 1:**
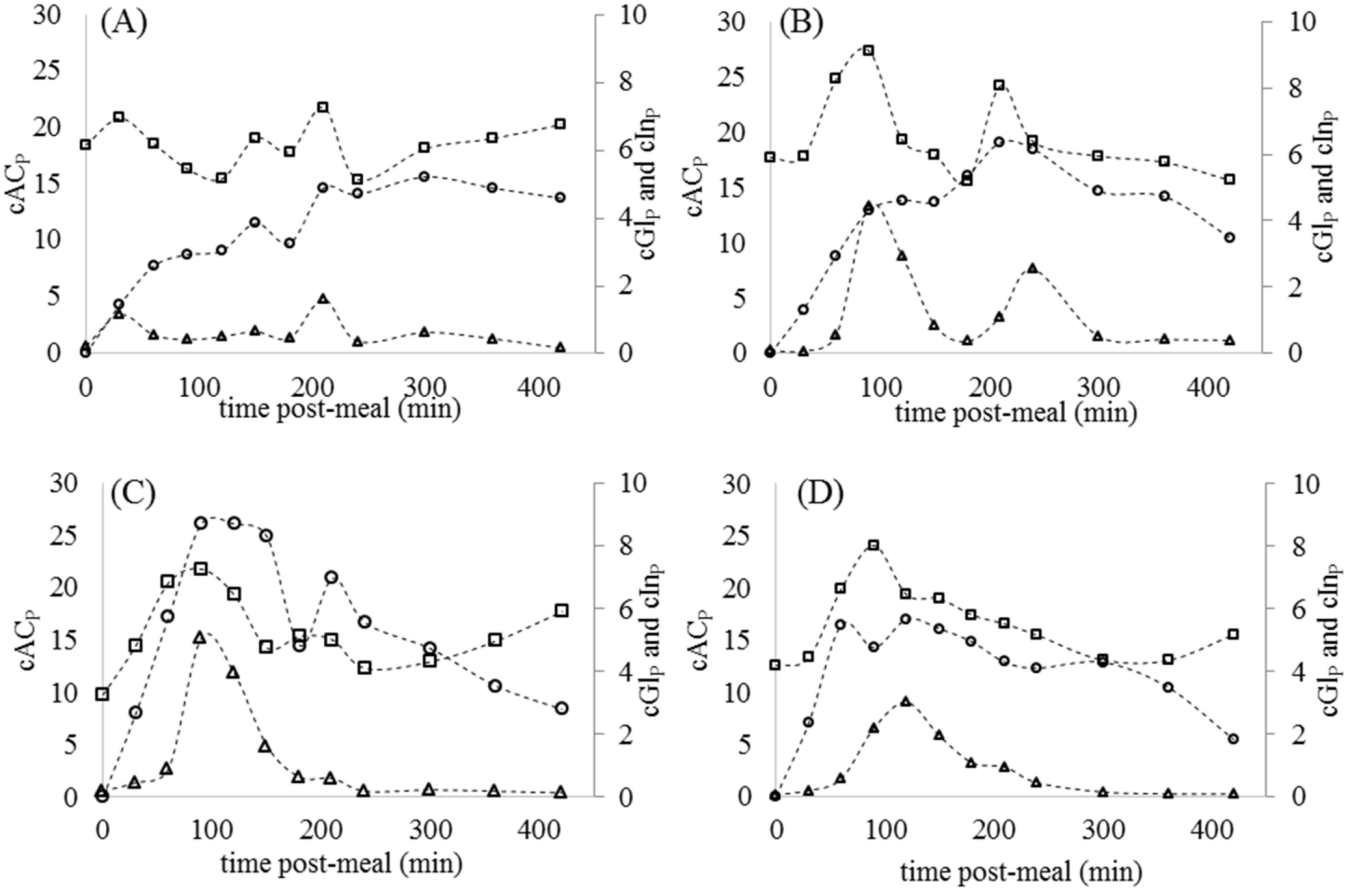
Observed postprandial plasma glucose (cGl_P_—square, mM), insulin (cIn_P_−triangle, ng/ml) and acetaminophen (cAC_P_−circle, mg/ml) relative to meal intake at time = 0 minutes for four representative data sets. (A) Animal 1. (B) Animal 2. (C) Animal 3. (D) Animal 4.

The animal datasets are shown in Fig 1. The timecourses of cGl_P_ and cIn_P_ do not all exhibit the ideal behaviour of an oral glucose tolerance test, where there is typically a rapid rise to a peak followed by a sustained decline back to baseline. Rather, the concentrations tend to fluctuate up and down throughout the 420 min, in delayed synchrony with changes in cAc_P_ that reflect gastric emptying rates, and may even sink below baseline concentrations prior to the meal. These erratic behaviours must be accommodated in the simulation model. All model parameters and descriptions are listed in Table 1.

**Table 1:** Variable descriptions and reference animal parameter values.

	Units	Ref animal	Description
**Animal**			
BW	kg	60	Body weight
iAc_S_	mg	3234	Dose of Ac administered orally (150 mg/kg BW^0.75^)
iAc_P_	mg	0	Basal plasma Ac mass prior to Ac bolus
iGl_S_	mmol	396	Dose of glucose present in meal
iGl_P_	mmol	90	Basal plasma glucose mass prior to meal
iIn_P_	mg	5.92	Basal plasma insulin mass prior to meal
iIs	mg	0.393	Basal insulin signal mass prior to meal
Ac_S_	mg	Variable	Stomach Ac mass
Ac_P_	mg	Variable	Plasma Ac mass
cAc_P_	mg L^-1^	Variable	Plasma Ac concentration
Gl_S_	mmol	Variable	Stomach glucose mass
Gl_P_	mmol	Variable	Plasma glucose mass
cGl_P_	mmol L^-1^	Variable	Plasma glucose concentration
In_P_	μg	Variable	Plasma insulin mass
cIn_P_	μg L^-1^	Variable	Plasma insulin concentration
Is	μg L^-1^	Variable	Insulin signal
**Acetaminophen**			
k_SP,2_	min^-1^	0.0015	First-order, slow gastric emptying rate constant
k_SP,3_	min^-1^	0.003	First-order, fast gastric emptying rate constant
k_Ac,UAc_	min^-1^	0.0022	First-order Ac utilization rate constant
Z	unitless	2,1,2,2,1,0,0,1,1,1,0,0,0,0	Z = 2,1, or 0 when gastric emptying is fast, slow, or not occurring, respectively
**Glucose**			
k_Gl,UGl_	L min^-1^	8.7e-7	First-order, glucose-dependent glucose utilization rate constant
k_Is,UGl_	L^2^ μg^-1^ min^-1^	0.0757	First-order, insulin-dependent glucose utilization rate constant
iPGl_end_	mmol min^-1^	0.178	Initial rate of endogenous glucose production
T_lag,SP_	min	15	Absorption lag time from stomach to plasma
**Insulin**			
K_Gl,Pin_	mmol L^-1^	8.8	Glucose-dependent insulin secretion Michaelis constant
k_In,UIn_	L min^-1^	0.7	First-order insulin utilization rate constant
V_Pin_	μg min^-1^	10	Maximal rate of insulin secretion
exp_Pin_	unitless	9	Hill coefficient for glucose-dependent insulin secretion
T_lag,Is_	min	16	Signaling lag time from In to Is

### Gastric Emptying

**Fig 2:**
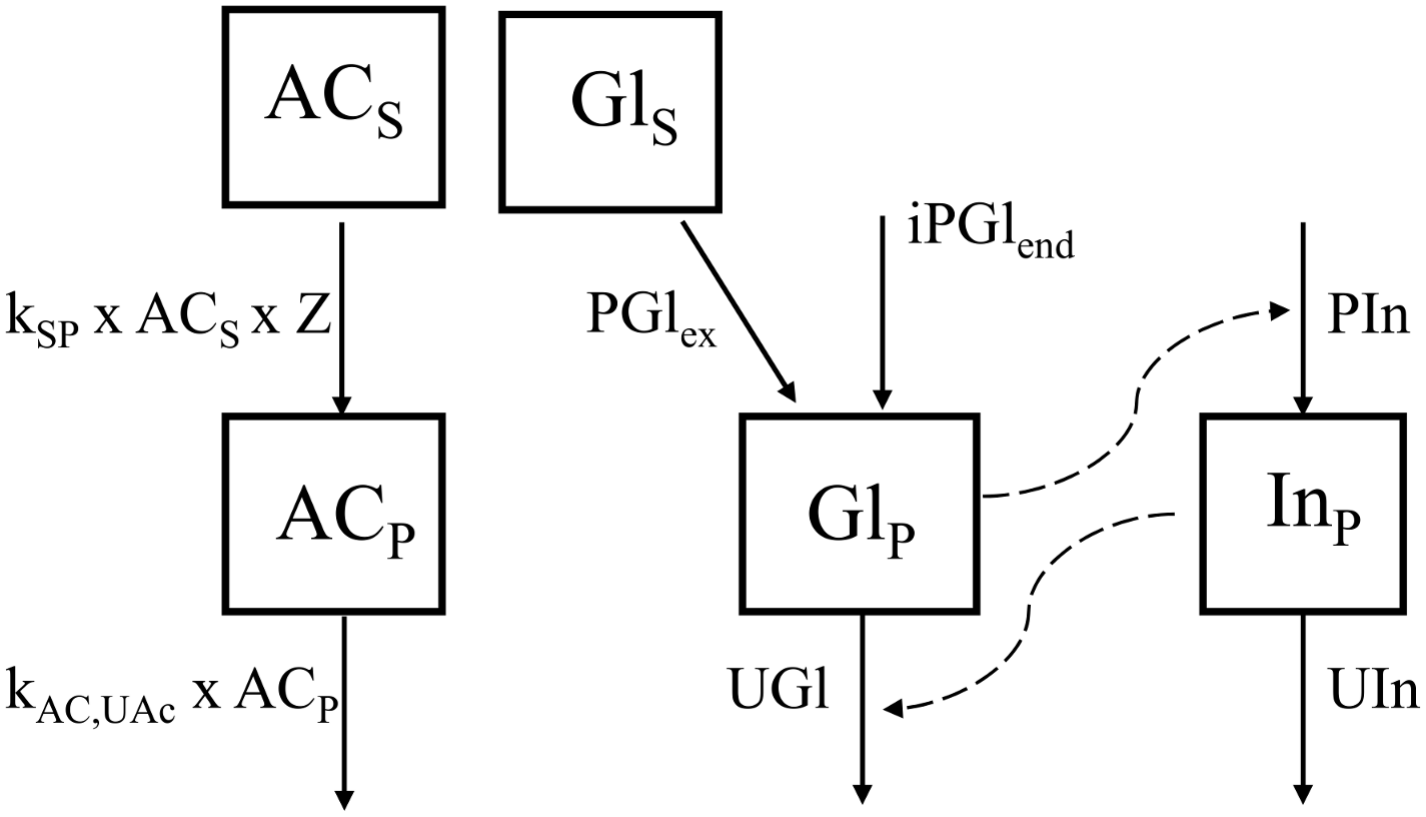
Schematic representation of a mechanistic model simulating intermittent gastric emptying and glucose-insulin dynamics. Solid arrows represent mass fluxes (see Table 1 for parameter descriptions); dashed lines represent stimulatory effects; the dotted line represents inhibitory effect; boxes represent state variables; Ac_S_ = stomach acetaminophen (mg), Ac_P_ = plasma acetaminophen (mg), Gl_S_ = stomach glucose (mmol), Gl_P_ = plasma glucose (mmol), In_P_ = plasma insulin (μg), k_SP_ = gastric emptying rate constant (min^-1^), k_Ac,UAc_ = first-order acetaminophen utilization rate constant (min^-1^), PGl_ex_ = rate of exogenous glucose appearance (mmol min^-1^),), iPGl_end_ = rate of endogenous glucose appearance (mmol min^-1^), PIn = rate of pancreatic insulin release (μg/min), UIn = rate of insulin utilization (μg/min).

To simulate gastric emptying of Ac in horses, Cant *et al*. [10] assumed that flow of digesta out of the stomach was either on or off during intervals between successive blood samples and multiplied the first-order rate constant for gastric emptying (k_SP_) by a Z-value of either 1 or 0 to indicate whether gastric emptying was occurring (ΔcAc_P,i_ > 0 in sampling interval i) or not (ΔcAc_P,i_ ≤ 0), respectively. We used the same approach, where initial mass of Ac in the stomach (iAc_S_ = initial Ac_S_) is given by the dose of Ac administered with the meal, and the rate of disappearance to blood follows first order kinetics (Fig 2):

$$\frac{{\text{dAc}}_{\text{S}}}{\text{dt}}=-{\text{k}}_{\text{SP}}\times {\text{Ac}}_{\text{S}}\times \text{Z}.$$ (1)

Plasma Ac (Ac_P_) arises from gastric emptying and disappears according to the first-order elimination constant (k_Ac,UAc_):

$$\frac{{\text{dAc}}_{\text{P}}}{\text{dt}}={\text{k}}_{\text{SP}}\times {\text{Ac}}_{\text{S}}\times \text{Z}-{\text{k}}_{\text{Ac,UAc}}\times {\text{Ac}}_{\text{P}}.$$ (2)

Volume of distribution of Ac_P_ was fixed at 0.9 L/kg BW [10], so that

$${\text{cAc}}_{\text{P}}=\frac{{\text{Ac}}_{\text{P}}}{0.9\text{BW}}.$$ (3)

Best-fit parameters of analytical solutions of the gastric emptying equations were estimated with the Solver function of Microsoft^®^ Office Excel^®^ 2007 to minimize residual sums of squares between predicted and observed cAc_P_. Curve fits were evaluated based on the root mean square prediction error (rMSPE) as a percentage of the mean, calculated as:

rMSPE%=∑i=1n(predi−obsi)2n∑i=1nobsin, (4)

where pred_i_ is the i-th prediction, obs_i_ is the i-th observation, and n is the number of observations.

**Fig 3:**
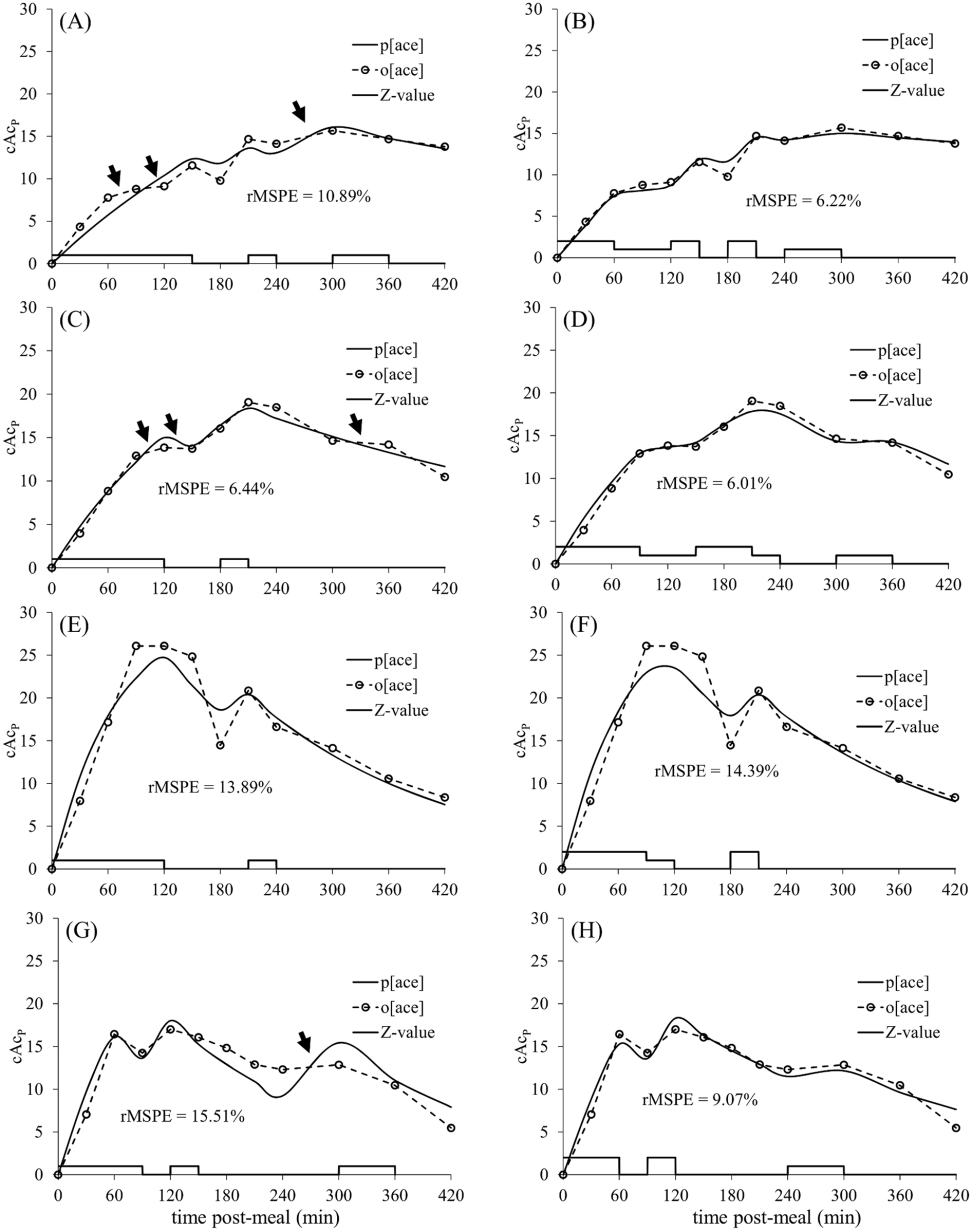
Observed (circles) and predicted (solid lines) post-prandial concentrations of plasma acetaminophen (cAc_P_; mg/ml) using 1 (A,C,E,G) or 2 (B,D,F,H) non-zero k_SP_ values for four test datasets. (A,B) Animal 1. (C,D) Animal 2. (E,F) Animal 3. (G,H) Animal 4. Arrows indicate where intermediate gastric outflow rates are apparent.

The model predicted cAc_P_ with an average rMSPE across all 4 calves of 12% of the mean observed cAc_P_. However, the model did not capture the nuances of apparently intermediate outflows between k_SP_ and 0 (arrows in Fig 3). Such intermediate flows were considered important to reproduce because of the changes in plasma glucose and insulin concentrations with which they were associated (Fig 1). An intermediate rate of Ac_P_ appearance indicates reduced or discontinuous outflow from the stomach during the sampling interval. This intermediate flow was accommodated with a second, non-zero k_SP_ value based on the slope (ΔcAc_P,_) between successive time points (i). Thus, k_SP_ = 0 when ΔcAc_P,i_ < -0.05 mg L^-1^ min^-1^, k_SP,2_ when -0.05 mg L^-1^ min^-1^ ≤ ΔcAc_P,i_ ≤ 0.05 mg L^-1^ min^-1^, and k_SP,3_ when ΔcAc_P,i_ > 0.05 mg L^-1^ min^-1^. The threshold value of 0.05 mg L^-1^ min^-1^ was chosen to minimize rMSPE. The parameter Z in eqs 1 and 2 was replaced with an array of Z-values equal to 0, 1 or 2 to denote k_SP_ = 0, k_SP,2_ or k_SP,3_, respectively, for each sampling interval (Fig 3). The prediction errors decreased from 12 to 9%, on average, when an intermediate gastric outflow was allowed (Fig 3).

### Glucose-Insulin Dynamics

Plasma glucose dynamics (Fig 2) are due to exogenous appearance (PGl_ex_) and endogenous production (PGl_end_) and utilization of glucose (UGl), so that

$$\frac{{\text{dGl}}_{\text{P}}}{\text{dt}}={\text{PGl}}_{\text{ex}}+{\text{PGl}}_{\text{end}}-\text{UGl}.$$ (5)

Gastric emptying rates k_SP,2_ and k_SP,3_ were used to represent emptying of carbohydrate in the meal from the stomach to the small intestine, as

$$\frac{{\text{dGl}}_{\text{S}}}{\text{dt}}=-{\text{k}}_{\text{SP}}\times {\text{Gl}}_{\text{S}}.$$ (6)

Acetaminophen appearance in plasma during the first 30 min after the meal was typically as rapid as its appearance in the next 30 min (Fig 1), indicating uninterrupted gastric emptying of the meal during the first 60 min. However, plasma glucose concentrations did not typically increase until after the 30-min sample (Fig 1), which could be due to retention of lactose in the stomach or an additional time period for intestinal carbohydrate hydrolysis and absorption. To simulate the delay between the deliveries of acetaminophen and glucose into the plasma, we assigned an absorption time-lag of T_lag,SP_ minutes to the gastric emptying, so that

$${\text{PGl}}_{\text{ex}}\text{(t)}={\text{k}}_{\text{SP}}\times {\text{Gl}}_{\text{S}}\text{(t}-{\text{T}}_{\text{lag,SP}}\text{)}.$$ (7)

To simulate basal cGl_P_ before the meal, when PGl_ex_ = 0, consideration was given to PGl_end_. It is known that PGl_end_ decreases as PGl_ex_ increases following a meal, due in part to the effects of insulin on gluconeogenesis and glycogenolysis. However, the PGl_end_-suppressing effects of insulin on cGl_P_ are indistinguishable from the UGl-stimulating effects of insulin on cGl_P_ so we chose to represent insulin effects on cGl_P_ via UGl, according to the established equations of the Bergman *et al*. [2] minimal model, and not via PGl_end_. Thus, PGl_end_ is maintained at a constant, zero-order flux equal to an initial PGl_end_ (iPGl_end_), and UGl is a function of cGl_P_ and the insulin signal (Is) according to a glucose effectiveness constant (k_Gl,UGl_) and an insulin sensitivity constant (k_Is,UGl_):

$$\text{UGl}={\text{k}}_{\text{Gl,UGl}}\times {\text{cGl}}_{\text{P}}+{\text{k}}_{\text{Is,UGl}}\times \text{Is}\times {\text{cGl}}_{\text{P}}.$$ (8)

The Is in MINMOD is a first-order delay of cIn_P_ [12]. This approach is adequate when cIn_P_ exhibits a single peak in the timecourse, but if cIn_P_ rises and falls multiple times following a meal, due to intermittent gastric emptying, the first-order delay removes much of the temporal variation in Is. To retain this temporal variation in Is in the current model, we represented the delay as a time lag of T_lag,IS_ min, so that

$$\text{Is(t)}={\text{cIn}}_{\text{P}}\text{(t}-{\text{T}}_{\text{lag,IS}}\text{)}.$$ (9)

Prior to T_lag,IS_, Is(t) is given an initial value (iIs) equal to the baseline cIn_P_.

The differential equation for In (Fig 2) contains the difference between insulin production (PIn) and utilization (UIn):

$$\frac{{\text{dIn}}_{\text{P}}}{\text{dt}}=\text{PIn}-\text{UIn}.$$ (10)

In MINMOD, PIn is a linear function of cGl_P_ above a certain threshold [12]. This structure accommodates, with 2 parameters, the quasi-exponential relation between cGl_P_ and pancreatic insulin release at the lower end of its sigmoidal relationship [17] but it introduces a breakpoint around the threshold cGl_P_ value that causes the first derivative to be discontinuous, which we considered undesirable for continuous simulations that may cross this threshold several times in one run. We chose to simulate sigmoidal kinetics of PIn relative to cGl_P_ continuously with the Hill equation of 3 parameters (V_PIn_, K_Gl,PIn_ and exp_PIn_):

$$\text{PIn}=\frac{{\text{V}}_{\text{PIn}}}{1+{\text{(}{\text{K}}_{\text{Gl,PIn}}/{\text{cGl}}_{\text{P}}\text{)}}^{{\text{exp}}_{\text{PIn}}}}.$$ (11)

UIn remains, as in MINMOD, a mass-action effect of cIn_P_ according to the first-order rate constant k_In,UIn_:

$$\text{UIn}={\text{k}}_{\text{In,UIn}}\times {\text{cIn}}_{\text{P}}$$ (12)

Concentrations of Gl_P_ and In_P_ are calculated assuming a volume of distribution equal to 0.251 L/kg BW, previously estimated in calves [18]:

$${\text{cGl}}_{\text{P}}=\frac{{\text{Gl}}_{\text{P}}}{0.251\text{BW}}$$ (13)

and

$${\text{cIn}}_{\text{P}}=\frac{{\text{In}}_{\text{P}}}{0.251\text{BW}}$$ (14)

### Behaviour and Sensitivity Analyses

**Fig 4:**
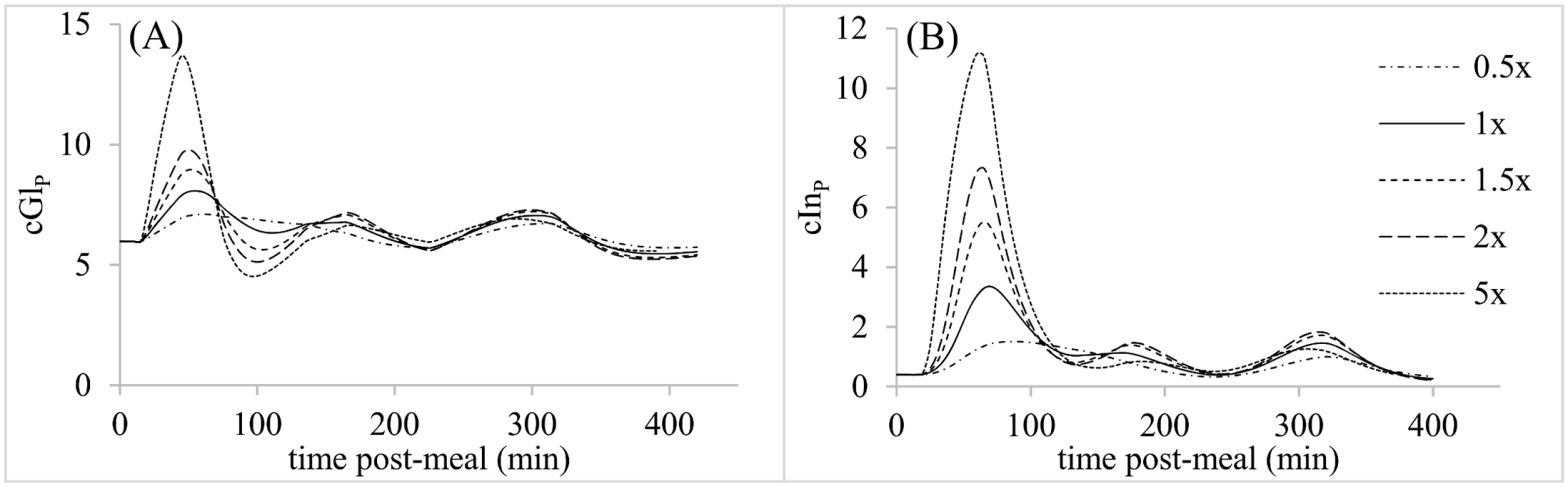
Parameter sensitivity analysis for postprandial first order gastric emptying rate while maintaining a constant ratio of k_SP,2_: k_SP,3;_; (A) Predicted plasma glucose concentrations (mM). (B). Predicted plasma insulin concentrations (ng/ml).

**Fig 5:**
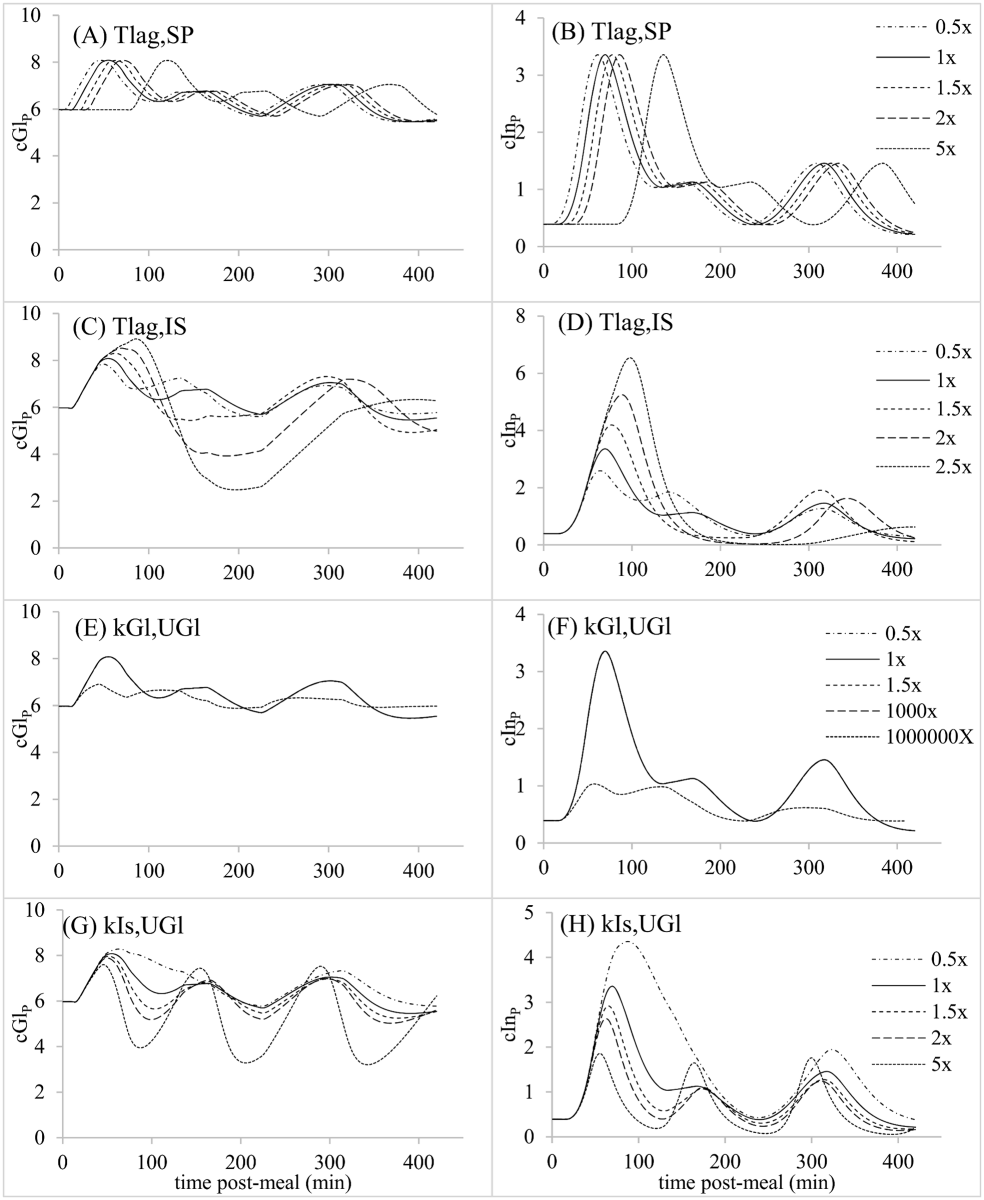
Parameter sensitivity analysis for parameters related to postprandial glucose dynamics; (A,C,E,G) Predicted plasma glucose concentrations (mM). (B,D,F,H). Predicted plasma insulin concentrations (ng/ml).

**Fig 6:**
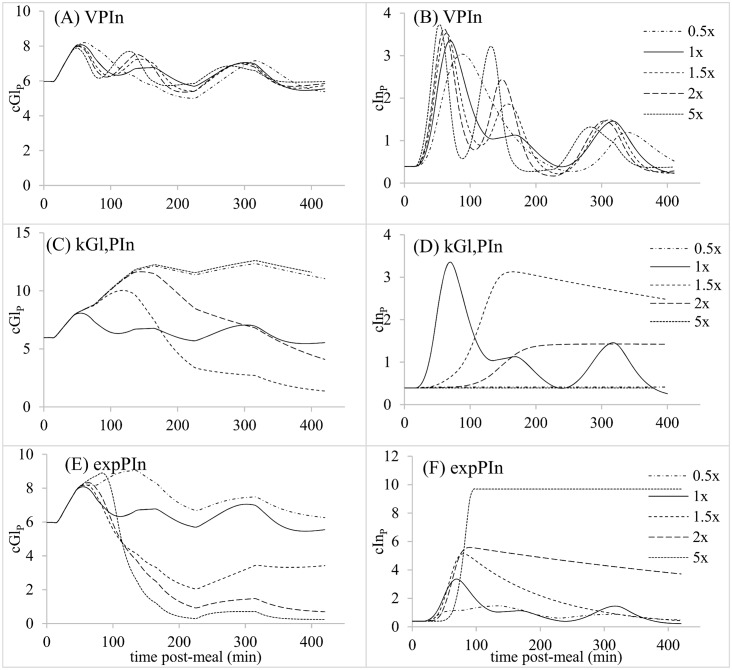
Parameter sensitivity analysis for parameters related to postprandial insulin dynamics; (A,C,E) Predicted plasma glucose concentrations (mM) (B,D,F). Predicted plasma insulin concentrations (ng/ml).

Differential equations of the model were written in ACSLX (Aegis Technologies Group, Inc., Orlando, USA) and solved with a 4^th^-order Runge-Kutta algorithm using an integration step size of 0.002 min. The final model contains 9 variables and 20 parameters (Table 1, S2 file), 8 of which can be obtained by direct measurement (BW, Z, iAc_S_, iAc_P_, iGl_S_, iGl_P_, iIn_P_ and iIs, where i represents the initial value) and 12 by fitting to observations (k_SP,2_, k_SP,3_, k_Ac,UAc_, k_Gl,UGl_, k_Is,UGl_, iPGl_end_, T_lag,SP_, K_Gl,PIn_, k_In,UIn_, V_PIn_, exp_PIn_ and T_lag,IS_). The model reproduces the multiple rises and falls in cGl_P_ and cIn_P_ that occur in calves during the 420 min following consumption of a normal-sized meal (Figs 4–6). Depending on parameter values, predicted cGl_P_ and cIn_P_ can fall below their respective baseline values prior to the meal, which is an important behaviour to reproduce as it is not uncommon (Fig 1). Values of the parameters can also affect the timing and degree of the cGl_P_ response to changes in gastric emptying and cIn_P_. In order to understand which characteristics of the postprandial glycemic response are affected by each of the parameters, they were each perturbed above and below reference values to an extent that allowed changes in the cGl_P_ and cIn_P_ curves to be examined. Reference values (Table 1) were set for a 60-kg calf consuming an Ac dose of 3234 mg and carbohydrate load of 396 mmol hexose-equivalents, representing a 2-L meal of milk replacer. The array of Z-values indicating fast, slow or zero gastric emptying was set to a typical pattern of fast initial emptying followed by intermittent, slow gushes. It was assumed that digestibility of lactose was 100% [19], 10% of intestinal galactose entered the circulation as free glucose [14,15] and 10% of intestinal glucose was removed by the splanchnic bed during absorption [7,9]. The remaining parameters of glucose-insulin dynamics were set to generate typical patterns of Ac, Gl and In appearance in plasma over the course of 420 min. Parameter assignments were subject to the constraint that differential eqs 5 and 10 equal zero at t = 0, so that the predicted non-steady, post-prandial state arises from a steady, pre-prandial state. Thus, when V_In_, K_Gl,PIn_, or exp_In_ were perturbed in the sensitivity analysis, k_In,UIn_ was set to

kIn,UIn=VPIniInP0.251BW[1+(KGl,PIniGlP0.251BW)expPIn], (15)

according to the steady-state constraint and eqs 10 to 14. Likewise, when k_Gl,UGl_ or k_Is,UGl_ were perturbed, the basal steady-state of Gl_P_ was achieved by setting

iPGlend=kGl,UGl×iGlP0.251BW+kIs,UGl×iInP0.251BW×iGlP0.251BW, (16)

according to eqs 5, 8, 9, 13 and 14.

The predicted reference postprandial patterns of cGl_P_ and cIn_P_ each exhibited 3 peaks in association with the gastric emptying profile (Figs 4 to 6). Altering T_lag,IS_, K_Gl,PIn_ and exp_PIn_ changed the number and amplitude of peaks in cGl_P_ and cIn_P_, while k_SP_, k_Gl,UGl_, k_Is,UGl_ and V_PIn_ only affected the amplitude of peaks (Figs 4 and 6). Of these latter parameters, k_Gl,UGl_ and V_PIn_ exerted relatively small effects on the amplitude of cGl_P_ peaks. In addition to affecting wave amplitudes, T_lag,IS_, k_Is,UGl_ and V_PIn_ shifted the times at which cGl_P_ or cIn_P_ peaks occurred. T_lag,SP_ also affected peak times without altering other characteristics of the glycemic response (Fig 5). According to these simulations, each of the parameters exerted unique effects on the postprandial responses, except for similarities between K_Gl,PIn_ and exp_PIn_, and k_Is,UGl_ and V_PIn_.

Gastric emptying is under neural and hormonal controls that regulate delivery of nutrients to the periphery for metabolism. How much of the glycemic response to a meal is due to gastric emptying rate versus pancreatic responsiveness and tissue insulin sensitivity remains an interesting question for the maintenance of normoglycemia and the attendant therapeutic implications. Because the model contains all three of these control elements, we used it to evaluate the relative role of each in the glycemic response, as indicated by areas under 420-min cGl_P_ and cIn_P_ curves (AUC_Gl_ and AUC_In_, respectively). Results of changing each parameter from 0.5 to 1.5X its reference value are presented as a sensitivity coefficient (SC_A_), equal to the fractional change in AUC_A_ relative to the fractional change in parameter value:

SCA=|AUCA(1.5x)-AUCA(0.5x)AUCA(1.5x)|((1.5-0.5)1.5). (17)

Parameters that affected AUC_Gl_ the most (Table 2) were those related to pancreatic response (K_Gl,PIn_ and exp_PIn_), followed by insulin sensitivity (k_Is,UGl_, T_lag,IS_) and then gastric emptying (k_SP_). The sensitivity to K_Gl,PIn_ was 50X that to k_SP_. However, sensitivity of AUC_In_ was 20X greater than sensitivity of AUC_Gl_ to k_SP_ (Table 2). Pancreatic responsiveness and insulin sensitivity parameters still exhibited stronger effects than the gastric emptying parameter on AUC_In_, but only by 2X instead of 50X. The difference between insulin and glucose responses to k_SP_ is interesting because one might intuitively presume that both would respond in a similar fashion, given the reciprocal nature of the control paradigm, in which cGl_P_ affects cIn_P_, and cIn_P_ affects cGl_P_. A large effect of k_SP_ on AUC_In_, with a much smaller effect on AUC_Gl_, suggests that pancreatic responsiveness is stimulated by faster gastric emptying, so that rapid insulin release (high AUC_In_) prevents hyperglycemia, leading to low AUC_Gl_. When gastric emptying was delayed in humans by the amylin analog pramlintide, AUC_In_ over the first 120 min decreased significantly while AUC_Gl_ over the same time period was affected little [20], similar to our simulation results. This decrease was not accompanied by a large change in pancreatic responsiveness parameters [20]. Our simulations show that the pancreatic response to k_SP_ can be independent of changes in the parameters of pancreatic responsiveness V_In_, K_Gl,PIn_ and exp_PIn_, because we found an increase in AUC_In_ without altering those parameters. The cause of the higher AUC_In_ as k_SP_ increased in our simulations was the quasi-exponential nature of the effect of cGl_P_ on insulin release, where small changes in cGl_P_ exert larger effects on PIn when cGl_P_ is at high values compared to low.

**Table 2:** Reference animal parameter sensitivity coefficients listed from highest to lowest influence on glucose area under the curve.

Parameter	Glucose	Insulin
K_Gl,PIN_	1.643	1.219
exp_Pin_	1.205	0.819
k_Is,UGl_	0.130	1.338
T_lag,IS_	0.052	0.124
k_SP_	0.030	0.618
V_PIn_	0.023	0.075
T_lag,SP_	0.004	0.010
k_Gl,UGl_	0.000	0.000

When k_SP_ was varied in the sensitivity analysis, amplitudes of peaks in cGl_P_ and cIn_P_ curves were affected but the number and timing of peaks did not change. Slower gastric emptying might be expected to delay the time to peak cGl_P_ and cIn_P_, as in the pramlintide experiment of Hinshaw et al. [20]. When gastric emptying was set at a continuous rate, by setting all instances of the parameter array Z = 2, then a slower k_SP_ prolonged the time to peak cIn_P_ (data not shown). Thus, time to peak is a consequence of the rate of gastric emptying (kSP × cGlS), and the number and sequence of fast versus slow or negligible gastric emptying bouts, which we did not present because of the large number of permutations possible.

### Parameter estimation

Estimation of k_SP,2_, k_SP,3_ and k_Ac,UAc_ from the 12 cAc_P_ data points has already been described. The remaining parameters of glucose-insulin dynamics (k_Gl,UGl_, k_In,UGl_, T_lag,SP_ and T_lag,IS_ in eqs 7 to 9, and V_PIn_, K_Gl,PIn_, exp_PIn_, and iIn_P_ in eqs 11, 12 and 14) were estimated with a differential evolution algorithm [21] to minimize the sum of residual sums of squares between predicted and observed cGl_P_, and predicted and observed cIn_P_. The differential evolution procedure is designed to search for optimal solutions within a large parameter space so that global rather than local minima in RSS are reached. A steady basal state was enforced with eqs 15 and 16. The evolutionary algorithm was run with 80 sets of parameter values in each generation. After 200 generations, means and standard errors of each parameter in the 30 best-fit sets were calculated.

**Fig 7:**
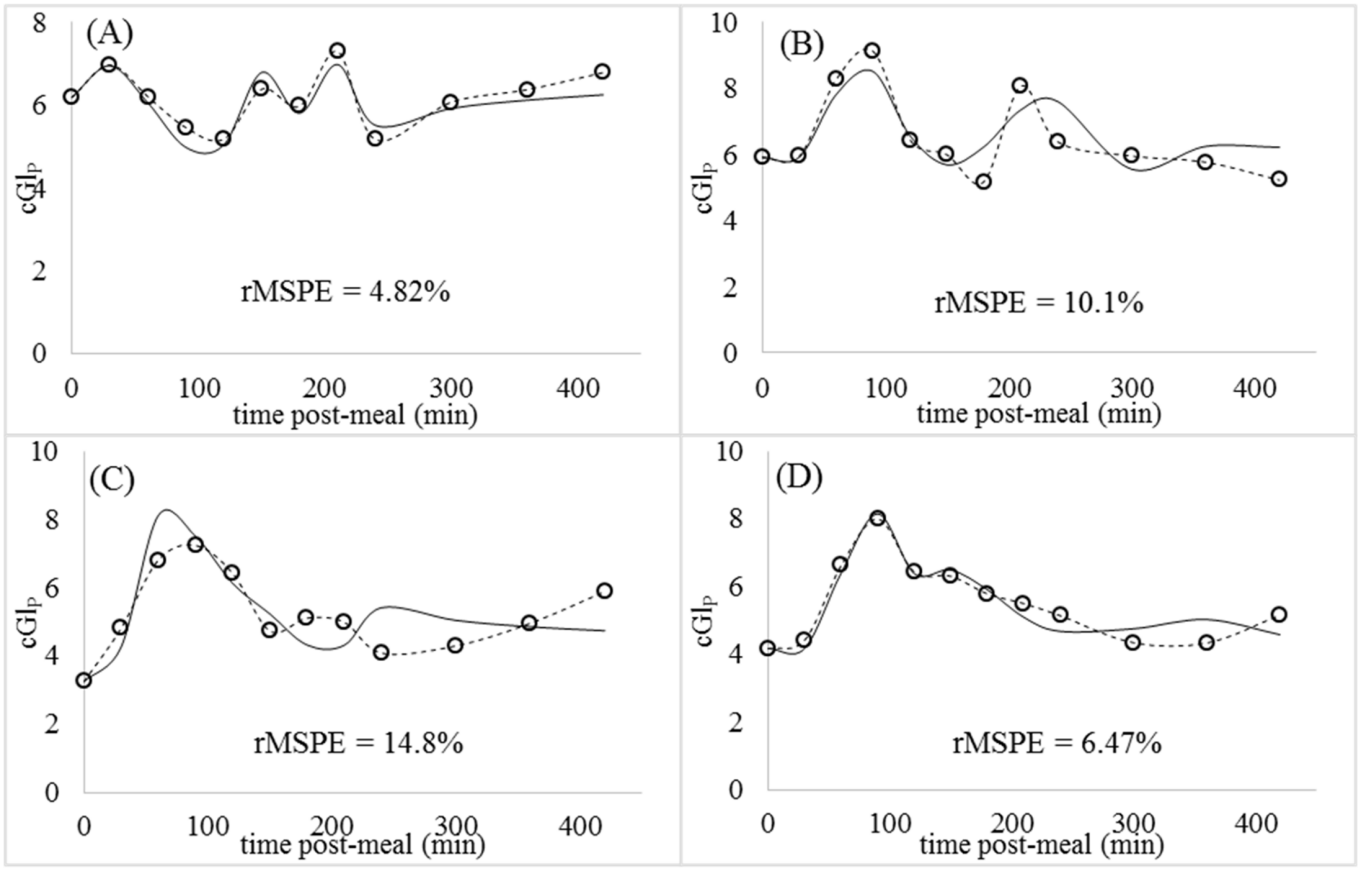
Observed (circles) and predicted (solid lines) postprandial plasma glucose (cGl_P_ -mM). (A) Animal 1. (B) Animal 2. (C) Animal 3. (D) Animal 4.

**Fig 8:**
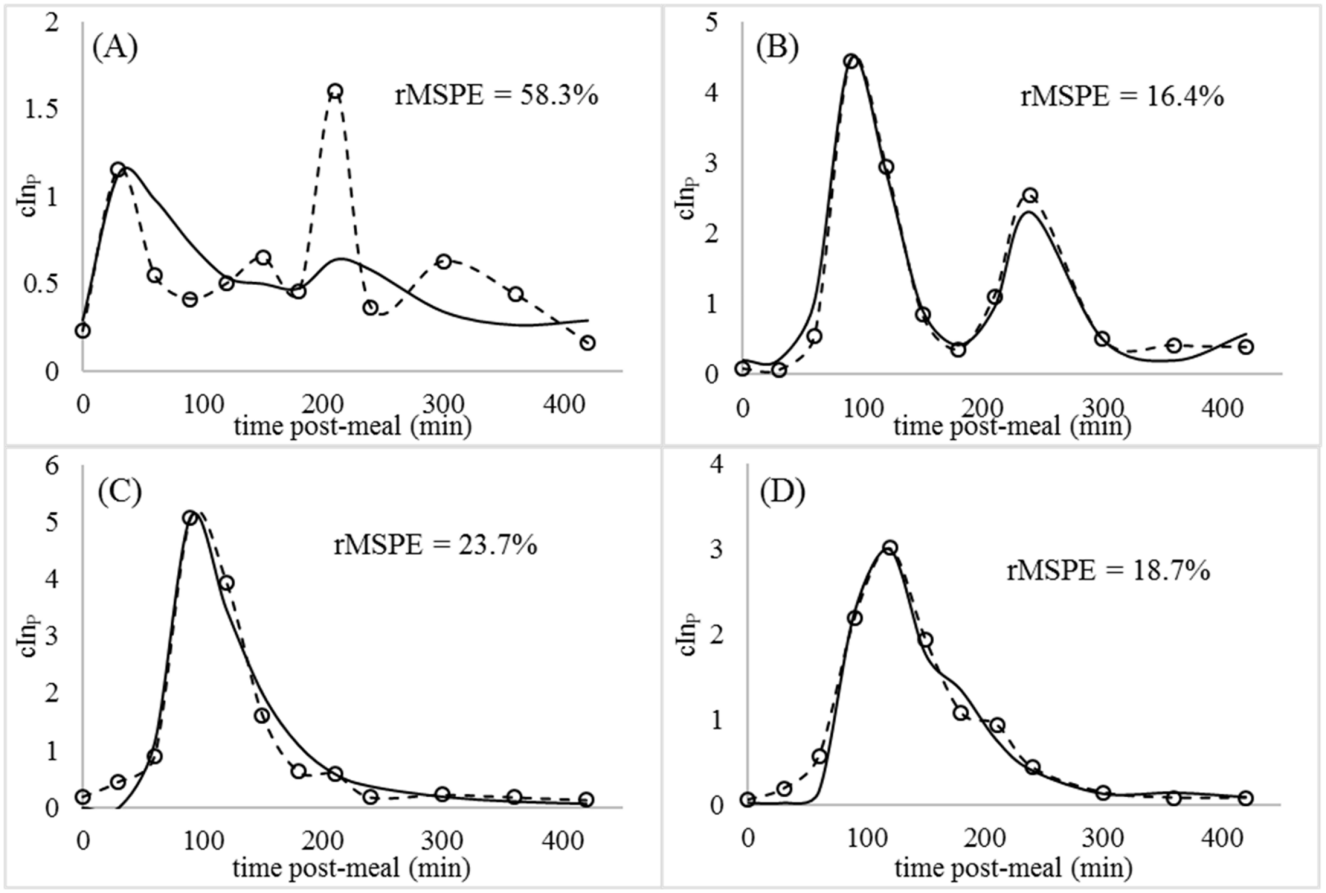
Observed (circles) and predicted (solid lines) postprandial plasma insulin (cIn_P_ -ng/ml). (A) Animal 1. (B) Animal 2. (C) Animal 3. (D) Animal 4.

Parameter estimates and their standard errors for each set of animal data are presented in S1 table. According to standard errors, parameter values were significantly different from 0 and were highly identifiable. The fitted aggregate model was able to reproduce postprandial glycemia in the 4 test subjects following the consumption of a milk-based meal with rMSPE percentages ranging from 5 to 15% for glucose (Fig 7) and from 19 to 58% for insulin (Fig 8). Curves with multiple rises and falls in cGlP and cInP were not worse fit than less erratic curves.

Entry rates of glucose into the plasma from intestinal lactose were based on fixed parameters of 100% absorption, 10% conversion of intestinal galactose to GlP, and 90% conversion of intestinal glucose to Gl_P_. To test the effect of these parameters on model outputs, we assessed fits to the 4 test subjects with 50% higher iGl_S_ values than what the fixed parameters predicted. Percentage rMSPE for cGl_P_ curves were 9.1% on average with the original iGl_S_ values and 9.5% with the higher iGl_S_ values, indicating little effect on curve fits.

## Discussion

We have presented a novel model for the simulation of postprandial glycemia based on intermittent, exponential gastric emptying with a time lag for appearance of intestinal glucose in plasma, and the minimal models of glucose and insulin dynamics of MINMOD [12]. Dalla Man *et al*. [8] accommodated intermittent gastric emptying in a model of postprandial glycemia as a convolution of three pulses with a second-order decay function for a total of 6 parameters. Other glycemia models with gastric emptying have assumed continuous gastric outflow, where the delay between emptying and appearance in plasma is acommodated with an intermediate intestinal pool from which absorption proceeds according to a first-order rate constant [7,9]. With 2 k_SP_ parameters and a lag-time (T_lag,SP_), our intermittent gastric emptying model of 3 parameters can be considered a minimal model.

We previously developed and assessed mathematical models of gastric emptying using data from horses given various dietary treatments containing Ac [10]. The best fit was achieved by a first-order model of plasma Ac appearance with parameters to describe the duration of periodic gushing of gastric contents as well as the quiescence between gushes. However, the use of two non-zero rate constants to describe gastric emptying rate in the current model provided a simpler solution for improvement of the prediction accuracy of Ac_P_ appearance. A comparison of Ac and Gl profiles in plasma indicated there was a delay between appearance of the liquid marker in the meal and appearance of glucose. Casein in the meal forms a clot in the stomach that slows its rate of emptying into the small intestine [22,23]. It is possible that the clot retains some of the liquid-associated lactose so that lactose entry into the small intestine is also slowed. However, cumulative bihourly samples of duodenal digesta collected from calves fed clotting or non-clotting milk replacers were not different in lactose content, indicating no effect of the clot on lactose emptying [22]. More frequent samples, as in our datasets [16], may be subject to a clot effect because the reference T_lag,SP_ values we found were less than 30 min. The contribution of lactose hydrolysis to the delay time does not appear to be significant because appearances of ^13^C label from intact and hydolyzed lactose in a meal consumed by lactose-tolerant humans were identical [24]. This leaves glucose absorption as a potential cause of the delay.

As a percentage of mean observations, prediction errors for cIn_P_ were higher than for cAc_P_ or cGl_P_. Part of the reason for higher relative errors is the large fold-changes in cIn_P_ that occur during the 420-min timecourse, compared to fold-changes in cAc_P_ and cGl_P_. However, the lower goodness of insulin fits suggests that improvements are possible in the insulin simulations, possibly through consideration of incretin dynamics. Although the model was developed using datasets from calves fed milk, it has utility for predicting post-meal glucose and insulin kinetics in other animal models as well as human subjects.

## Conclusion

The combination of minimal models of gastric emptying, plasma glucose dynamics and plasma insulin dynamics suitably describes the erratic postprandial glycemia following a milk-based meal, including depressions below the baseline cGl_P_ prior to the meal. Sensitivity analysis of the model indicates that faster gastric emptying increases pancreatic responsiveness and keeps plasma glucose concentrations low, independent of the parameters of pancreatic response, simply through the quasi-exponential nature of the relationship between cGl_P_ and pancreatic insulin release. The model has the potential to be used in the evaluation of dietary treatments for their net effects on pancreatic responsiveness, insulin sensitivity and glucose effectiveness, as well as to predict glycemic responses to various normal-sized meals.

## Supporting Information

S1 File Postprandial plasma acetaminophen (mg/L), glucose (mM) and insulin (ng/ml) of four datasets used for model development. (XLSX)

S2 File The acslX program with reference animal parameter values. (PDF)

S1 Table Parameters values of four test datasets. Best-fit parameters are shown as mean ± standard error.
(PDF)

## References

[1] Wolever TMS, Jenkins DJA, Jenkins AL, Josse RG. The glycemic index: methodology and clinical implications. Am J Clin Nutr. 1991;54:846–854. 1951155 10.1093/ajcn/54.5.846

[2] Bergman RN, Ider YZ, Bowden CR, Cobelli C. Quantitative estimation of insulin sensitivity. Am J Physiol. 1979;236:667–677. 10.1152/ajpendo.1979.236.6.E667 443421

[3] Toffolo G, Bergman RN, Finegood DT, Bowden CR, Cobelli C. Quantitative estimation of beta cell sensitivity to glucose in the intact organism. Diabetes. 1980;29:979–990. 7002673 10.2337/diab.29.12.979

[4] Janssen P, Vanden Berghe P, Verschueren S, Lehmann A, Depoortere I, Tack J. Review article: the role of gastric motility in the control of food intake. Aliment Pharmacol Ther. 2011;33:880–894. doi: 10.1111/j.1365-2036.2011.04609.x 21342212 10.1111/j.1365-2036.2011.04609.x

[5] Vist GE, Maughan RJ. The effect of osmolality and carbohydrate content on the rate of gastric emptying of liquids in man. J Physiol 1995;486:523–531. 7473216 10.1113/jphysiol.1995.sp020831 PMC1156540

[6] Dedik L, Durisova M, Penesova A, Miklovicova D, Tvrdonova M. Estimation of influence of gastric emptying on shape of glucose concentration-time profile measured in oral glucose tolerance test. Diabetes Res Clin Pr. 2007;77:377–384. 10.1016/j.diabres.2006.12.017 17270310

[7] Burattini R, Morettini M. Identification of an integrated mathematical model of standard oral glucose tolerance test for characterization of insulin potentiation in health. Comput Meth Prog Bio. 2012;107:248–261. 10.1016/j.cmpb.2011.07.002 21803437

[8] Dalla Man C, Caumo A, Cobelli C. The oral glucose minimal model: Estimation of insulin sensitivity from a meal test. IEEE Bio-Med Eng. 2002;49:419–429. 10.1109/10.995680 12002173

[9] Dalla Man C, Rizza RA, Cobelli C. Meal simulation model of the glucose-insulin system. IEEE Bio-Med Eng. 2007;54:1740–1749. 10.1109/TBME.2007.893506 17926672

[10] Cant JP, Walsh VN, Geor RJ. Obtaining information on gastric emptying patterns in horses from appearance of an oral acetaminophen dose in blood plasma In: Kebreab E., Dijkstra J., Bannink A., Gerrits W. J. J., France J. (eds), Nutrient Digestion and Utilization in Farm Animals: Modelling Approaches, CABI Publishing, Wallingford, UK; 2005 pp. 69–83

[11] Clements JA, Heading RC, Nimmo WS, Prescott LF. Kinetics of acetaminophen absorption and gastric emptying in man. Clin Pharmacol Ther 1978;24:420–431. 688732 10.1002/cpt1978244420

[12] Pacini G, Bergman RN. MINMOD: a computer program to calculate insulin sensitivity and pancreatic responsiveness from the frequently sampled intravenous glucose tolerance test. Comput Meth Prog Biomed. 1986;23:113–122. 10.1016/0169-2607(86)90106-9 3640682

[13] Bell FR, Razig SAD. Gastric emptying and secretion in the milk-fed calf. J Physiol. 1973;228:499–512. 4568910 10.1113/jphysiol.1973.sp010096 PMC1331307

[14] Gannon MC, Khan MA, Nuttall FQ. Glucose appearance rate after the ingestion of galactose. Metabolism. 2001;50:93–98. 11172481 10.1053/meta.2001.19442

[15] Barosa C, Silva C, Fagulha A, Barros L, Caldeira MM, Carvalheiro M et al Sources of hepatic glycogen synthesis following a milk-containing breakfast meal in healthy subjects. Metabolism. 2012;61:250–254. doi: 10.1016/j.metabol.2011.06.022 21862086 10.1016/j.metabol.2011.06.022

[16] MacPherson JAR, Berends H, Neal LN, Cant JP, Martin-Tereso J, Steele MA. Effect of plane of nutrition and age on glucose and insulin kinetics and abomasal emptying in female Holstein Friesian dairy calves fed twice daily. J Dairy Sci. In Press 10.3168/jds.2015-10826 27522426

[17] Malaisse WJ. Insulin secretion: Multifactorial regulation for a single process of release. Diabetologia.1973;9:167–173. 4368828 10.1007/BF01219778

[18] Benshop DL, Cant JP. Developmental changes in clearance of intravenous doses of glucose, acetate and β-hydroxybutyrate from plasma of calves. Livest Sci. 2009;122:177–185.

[19] Xu C, Wensing T, Beynen AC. High intake of calcium formate depresses macronutrient digestibility in veal calves fed milk replacers containing either dairy proteins or whey protein plus soya protein concentrate. J Anim Physiol Anim Nutr. 2000;83:49–54.

[20] Hinshaw L, Schiavon M, Mallad A, Dalla Man C, Basu R, Bharucha AE et al Effects of delaying gastric emptying on postprandial glucose kinetics, insulin sensitivity and β-cell function. Am J Physiol. 2014;307:E494–E502. 10.1152/ajpendo.00199.2014 PMC4166717 25074985

[21] Storn R, Price K. Differential Evolution–A simple and efficient heuristic for global optimization over continuous spaces. J Global Optim. 1997;11:341–359.

[22] Petit HV, Ivan M, Brisson GJ. Duodenal flow of digesta in preruminant calves fed clotting or nonclotting milk replacer. J Dairy Sci. 1987;70:2570–2576. 3448108 10.3168/jds.S0022-0302(87)80326-0

[23] Le Huërou-Luron I, Gestin M, Le Dréan G, Romé V, Bernard C, Chayvialle JA et al Source of dietary protein influences kinetics of plasma gut regulatory peptide concentration in response to feeding in preruminant calves. Comp Biochem Physiol. 1998; 119A:817–824. 10.1016/s1095-6433(98)01021-6 9683415

[24] Stellaard F, Koetse HA, Elzinga H, Boverhof R, Tjoonk R, Klimp A et al 13C-Carbohydrate breath tests: impact of physical activity on the rate-limiting step in lactose utilization. Scand J Gastroenterol. 2000;35:819–823. 10994620 10.1080/003655200750023183

