# Supplementary material for: A Mechanistic Model of Intermittent Gastric Emptying and Glucose-Insulin Dynamics following a Meal Containing Milk Components
Source: PLoS One. 2016 Jun 2;11(6):e0156443. doi: 10.1371/journal.pone.0156443 (PMC4890795; doi:10.1371/journal.pone.0156443)
Supplement: S2 File — (PDF) [file pone.0156443.s002.pdf]

```
PROGRAM aceglcins
INITIAL ! units are dose mg, min, blood Ac mg/L, BW kg
        CONSTANT iACs=3234, kAcUAc=0.0022, tstp=420
        CONSTANT iACp=0.0000001, BW=60, iGLs=396
        CONSTANT iGLp=90
! code to set CINT to either 30 or 60 min to match with observations
  INTEGER phase
  phase=1
  DIMENSION CINTTAB(2)
  CONSTANT CINTTAB=30, 60
! code to switch gastric emptying on and off
! z-values every 30 min indicate whether GE on slow(1), on fast(2) or off(0) based on chg in [acetamin]
  INTEGER sampnos
  INTEGER z
  DIMENSION z(14)
  CONSTANT z=2,1,2,2,1,0,0,1,1,1,0,0,0,0
! kSP(1) when z=0, kSP(2) when z=1, kSP(3) when z=2
  INTEGER kSPcntr
  DIMENSION kSP(3)
  CONSTANT kSP = 0,0.0015,0.003
END ! of initial
DYNAMIC
        ALGORITHM IALG=5
        NSTEPS NSTP=1
        MAXTERVAL MAXT=.002
DERIVATIVE
        TERMT (T.GE.TSTP)
        PROCEDURAL (phase=t) ! to match output times with observed sampling times
                IF (T.GT.230) phase=2
        END ! of PROCEDURAL
        sampnos=INT(t/30.0)+1
        dACsdt=-absAc
                kSPcntr=z(sampnos)+1
                absAc=kSP(kSPcntr)*ACs
        ACs=INTEG(dACsdt, iACs)
        dACpdt=absAc-kAcUAc*ACp
        ACp=INTEG(dACpdt, iACp)
        cACp=ACp/(BW*0.9)

       dGLsdt=-absGl
                absGl = kSP(kSPcntr)*GLs
        GLs=INTEG(dGLsdt, iGLs)

! glucose/insulin dynamic model
```

```
! with insulin signalling pool

CONSTANT TlagSP=15 ! can't be larger than 60 without changing delay function
CONSTANT KGlUGl=0.0000087, KIsUGl=0.0757
CONSTANT iPGLend=0.178
        dGLpdt = PGLex + PGLend - UGl
              PGLex = DELAY(absGl, 0, TlagSP, 100000) ! absorption lag-time
              PGLend=iPGLend
              UGl=kGlUGl*cGLp+kIsUGl*Is*cGLp
        GLp = INTEG(dGLpdt, iGLp)
        cGLp = GLp/(BW*0.251)

       Is=DELAY(cINp, iIs, TlagIS, 50000)

        AUCGl=INTEG(cGLp-iGLp/(BW*0.251),0)

CONSTANT expPIn=9, iINp=5.92, kInUIn=0.7
CONSTANT KGlPIn=8.8, VPIn=10, iIs=0.393, TlagIS=16
        dINpdt = PIn - UIn
             PIn = VPIn/(1+(KGlPIn/cGLp)**expPIn)
             UIn = kInUIn*cINp
        INp = INTEG(dINpdt, iINp)
        cINp = INp/(BW*0.251)

        AUCINp=INTEG(cINp-iINp/(BW*0.251),0)

END ! of derivative
        CINT=CINTTAB(phase)
END ! of dynamic
END ! of program
```
