# Supplementary material for: A Mechanistic Model of Intermittent Gastric Emptying and Glucose-Insulin Dynamics following a Meal Containing Milk Components
Source: PLoS One. 2016 Jun 2;11(6):e0156443. doi: 10.1371/journal.pone.0156443 (PMC4890795; doi:10.1371/journal.pone.0156443)
Supplement: S1 Table — Best-fit parameters are shown as mean ± standard error. (PDF) [file pone.0156443.s003.pdf]

	Units	Animal 1	Animal 2	Animal 3	Animal 4
**Animal**					
BW	kg	75.1	87.7	77.8	68.4
iAc_S	mg	3827	4299	3929	3568
iGl_S	mmol	793	793	396	396
iGl_P	mmol	116	130	63.6	71.8
iIn_P	µg	5.40 ± 0.002	6.16 ± 0.016	0.033 ± 3.8e-4	0.787 ± 0.02
**Acetaminophen**					
$k_{SP,2}$	min^{-1}	0.00055	0.00179	0.00484	0.00175
$k_{SP,3}$	min^{-1}	0.00240	0.00360	0.00792	0.00588
$k_{Ac,UAc}$	min^{-1}	0.00060	0.00333	0.00452	0.00387
Z	unitless	2,2,1,1,2,0,2, 0,1,1,0,0,0,0	2,2,2,1,1,2,2, 1,0,0,1,1,0,0,	2,2,2,1,0,0,2, 0,0,0,0,0,0,0	2,2,0,2,0,0,0, 0,1,1,0,0,0,0
**Glucose**					
$k_{Gl,UGl}$	L min^{-1}	0.194 ± 0.0001	0.616 ± 0.0011	1.67e-3 ± 2.6e-4	0.18 ± 0.002
$k_{Is,UGl}$	L^2 μg^{-1} min^{-1}	0.432 ± 0.0002	0.101 ± 1.8e-4	0.082 ± 9.8e-5	0.04± 4.7e-4
$iPGl_{end}$	mmol min^{-1}	1.96	3.80	5.85e-3	0.79
$T_{lag,SP}$	min	0.004 ± 0.0001	40.13 ± 0.032	23.8 ± 0.04	40.4 ± 0.09
**Insulin**					
$K_{Gl,PIn}$	mmol L^{-1}	10.35 ± 0.005	10.28 ± 0.015	12.84 ± 0.06	17.8 ± 0.13
$k_{In,UIn}$ (calculated)	L min^{-1}	0.197	1.04	0.470	0.66
V_{Pin}	µg min^{-1}	391.6 ± 2.86	37.72 ± 0.34	268.7 ± 9.07	842.8 ± 17.8
exp_{PIn}	unitless	17.16 ± 0.004	8.81 ± 0.012	9.37 ± 0.012	7.31 ± 0.05
$T_{lag,IS}$	min	0.0001 ± 1.6e-5	22.15 ± 0.037	0.12±0.023	1.10 ± 0.16
